# Vanishing Bones and Stubborn Joints: Unravelling the Enigma of Multicentric Osteolysis, Nodulosis, and Arthropathy Syndrome

**DOI:** 10.7759/cureus.94584

**Published:** 2025-10-14

**Authors:** Rajat Kumar Sahu, Mukesh Maurya, Monalisha Sahoo, Urmila Dhakad, Puneet Kumar

**Affiliations:** 1 Clinical Immunology and Rheumatology, King George's Medical University, Lucknow, IND; 2 Research, King George's Medical University, Lucknow, IND

**Keywords:** and arthropathy (mona), arthritis, mmp 2, mona, multicentric osteolysis, nodulosis, osteolysis

## Abstract

Multicentric osteolysis, nodulosis, and arthropathy (MONA) syndrome is a rare inherited skeletal disorder, transmitted in an autosomal recessive manner, marked by gradual bone loss, especially affecting the carpal and tarsal bones, and the presence of subcutaneous nodules. The condition results from mutations in the *MMP2* gene, which encodes matrix metalloproteinase-2, an enzyme essential for extracellular matrix remodeling. MONA typically begins in early childhood and can resemble juvenile idiopathic arthritis, often leading to delayed or incorrect diagnosis.

We describe the case of two patients with progressive, noninflammatory joint deformities and characteristic palmoplantar nodules with coarse facies. Routine investigations were normal; X-rays of the affected joints showed lysis of multiple carpal, metacarpal, tarsal, and metatarsal bones. Therefore, whole exome sequencing was performed for diagnosis. We analyzed pertinent literature outlining the diverse clinical manifestations of MONA syndrome and emphasizing the pivotal importance of genetic testing in its diagnosis and management. Genetic testing revealed homozygous *MMP2* mutations, c.301C>T (p.Arg101Cys) and c.556G>C (p.Gly186Arg), confirming the diagnosis of MONA syndrome. Our literature review included 12 relevant articles outlining clinical features and associated genetic mutations. Early suspicion of MONA is crucial to prevent inappropriate use of immunosuppressants and to facilitate timely genetic confirmation. Given the condition’s rarity and clinical overlap with other disorders, case reports are the key to advancing diagnosis and further supportive management.

## Introduction

Multicentric osteolysis, nodulosis, and arthropathy (MONA), also referred to as Torg-Winchester syndrome, is an uncommon and disabling skeletal condition marked by advancing bone resorption, joint deformities, subcutaneous nodules, gum hypertrophy, and eye, skin, and heart anomalies [1]. Osteolysis mostly involves carpal and tarsal bones. The two genes affected are the* MMP2* and *MMP14* genes. Both genes are expected to generate phenotypic variations of the same disease. Pathogenic variants disrupt regular collagen metabolism and lead to a loss of gelatin-degrading activity of the metalloproteinase enzyme [2].

The hallmark skeletal features are osteolysis of carpal and tarsal bones with progressive deformity associated with subcutaneous nodules [3,4]. Flexion deformities of the proximal interphalangeal (PIP) joints (camptodactyly) are most prominent in the hands and progressively involve larger joints, leading to increasing functional impairment. A similar progression is typically observed in the feet [5,6]. Non-skeletal manifestations may include facial dysmorphism, gingival hypertrophy, high arched palate, delayed tooth eruption, subcutaneous nodules, hypertrichosis, corneal opacity, and congenital cardiac defect [7,8]. The rarity of MONA syndrome cases continues to obscure a clear definition of its full phenotypic landscape [9,10]. Less than 50 cases of MONA syndrome have been reported worldwide in different populations. MONA syndrome has multiple differential diagnoses, including Ter Haar syndrome (homozygous mutation in the *TKS4* gene) [11], multicentric carpotarsal osteolysis syndrome [12], Winchester syndrome [13], juvenile idiopathic arthritis, and mucopolysaccharidosis type I as Scheie syndrome [14].

This case report contributes to the understanding of MONA through two detailed cases, highlighting its clinical, radiological, and genetic features. It underscores diagnostic challenges, restricted therapeutic choices, and the necessity for a collaborative, multi-specialty approach involving rheumatology, orthopedics, genetics, and physiotherapy to improve patient outcomes. Parental informed consent was secured prior to publication, upholding ethical standards and safeguarding the child’s medical confidentiality throughout the reporting process.

## Case presentation

Case 1

**Figure 1:**
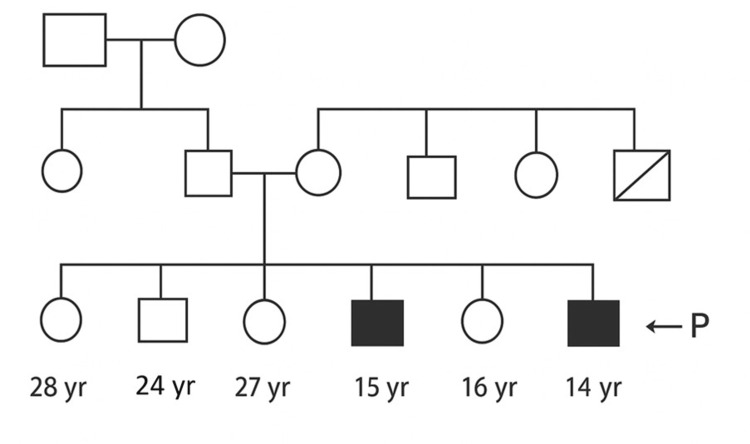
Pedigree analysis of the first patient

A 14-year-old boy, with no parental consanguinity, reported a slow and progressive onset of joint pain, swelling, and deformities involving both small and large joints, without any morning stiffness, since six years of age. Initial symptoms began in the left knee and progressively involved other joints over five months, with deformities developing over the next two years. These joint deformities severely impacted his day-to-day activities, making it difficult for him to walk, hold objects, or carry out basic tasks such as mixing food independently. He also noted the appearance of firm nodular swellings over his PIP joints, wrists, knees, and ankles. Despite various over-the-counter medications, joint swelling and deformities persisted. There was no history of skin rash, uveitis, visual disturbance, or back pain. Developmental milestones were normal. His elder brother had similar nodular swellings and joint deformities (Figure 1).

**Figure 2:**
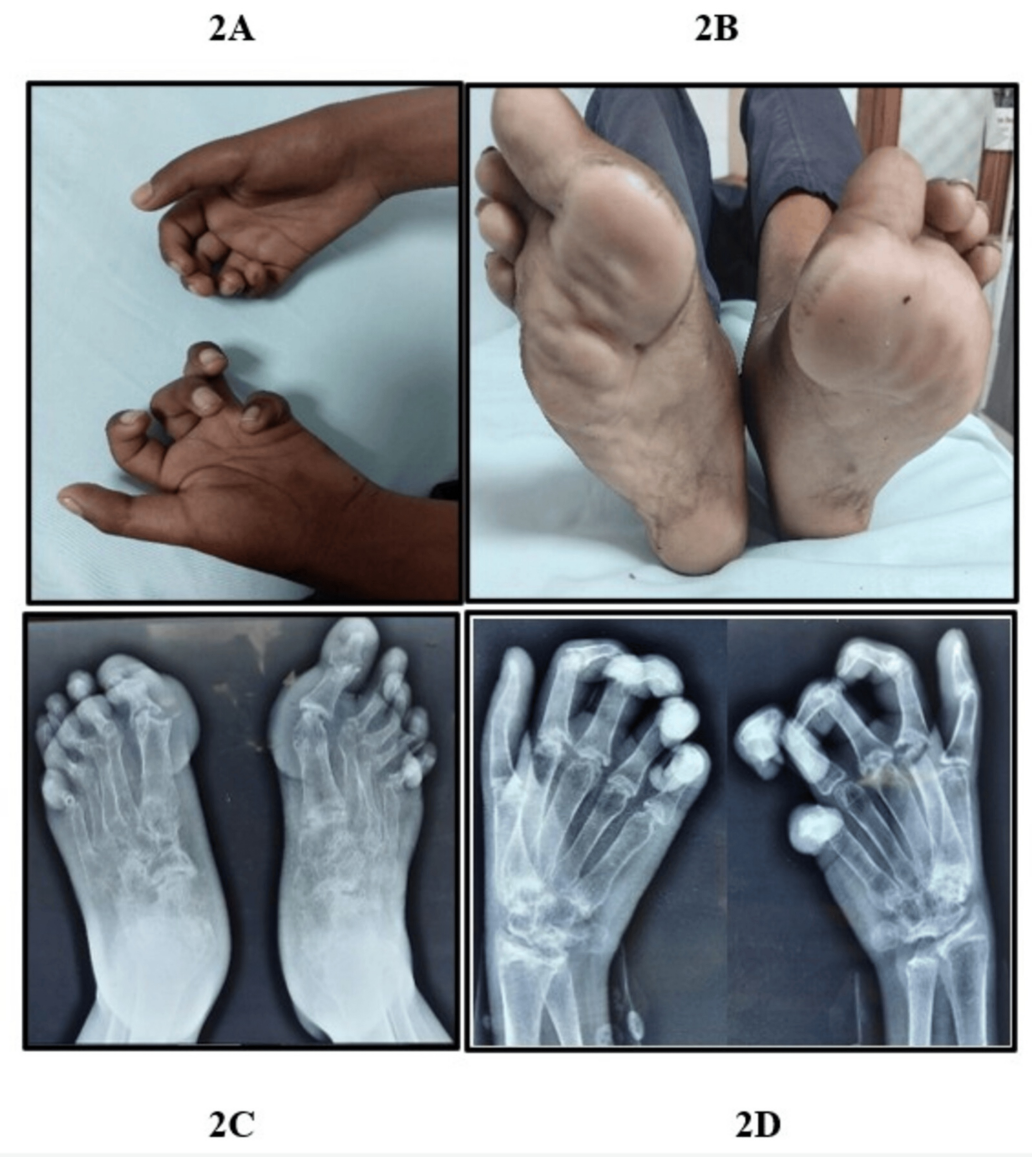
(A) Contracture of PIP and DIP joints of patient 1. (B) Nodular swelling on the bilateral plantar surface and overriding of little finger. (C) Lysis of multiple tarsal bones, extension deformity of MTP, and flexion deformity of IP. (D) Lysis of multiple carpal bones and flexion deformity of bilateral PIP and DIP with diffuse osteopenia. DIP, distal interphalangeal joint; IP, interphalangeal joint; MTP, metatarsophalangeal joint; PIP, proximal interphalangeal joint

Clinical examination revealed marked flexion contractures involving the PIP and distal interphalangeal (DIP) joints of both hands and feet, giving rise to noticeable joint deformities (Figure 2A), accompanied by swelling in the wrists, knees, and midfoot. Firm subcutaneous nodules were palpated along the plantar surfaces bilaterally, with the largest measuring around 3 x 3 x 1 cm³ (Figure 2B). Tenderness was noted in multiple joints, including both knees, ankles, and metatarsophalangeal (MTP) joints. Notably, spinal and hip evaluations revealed no abnormalities.

Laboratory workup showed a hemoglobin level of 12.2 g/dL and a total leukocyte count of 5,431/mm³. Inflammatory indicators, including erythrocyte sedimentation rate (ESR) and C-reactive protein (CRP), along with autoantibodies, including rheumatoid factor (RF), anti-cyclic citrullinated peptide (anti-CCP), and anti-nuclear antibody (ANA), were within normal ranges, supporting a non-inflammatory origin for the symptoms. Radiographs demonstrated osteolysis of multiple tarsal bones with contracture of tarsometatarsal joints with soft tissue swelling (Figure 2C) and osteolysis of multiple carpal bones with contracture of PIP and DIP joints (Figure 2D). A homozygous pathogenic variant was identified in exon 2 of the *MMP*2 gene [c.301C>T; p.Arg101Cys], establishing the diagnosis of MONA syndrome.

Case 2

A six-year-old boy, born to parents with no consanguinity, was referred to our rheumatology department with a 1.5-year complaint of progressively worsening symptoms and non-painful joint swelling and deformities, affecting multiple small and large joints symmetrically with no morning stiffness. Ankles were initially involved, which rapidly progressed and involved several other joints within a short three-month period. She reported difficulty in walking, grasping objects, and mixing food, with no improvement despite over-the-counter medications. There was no prior history of psoriasis, eye inflammation, inflammatory back pain, recent infections, or familial joint diseases. The child’s developmental milestones were appropriate for age, and immunizations were up to date.

**Figure 3:**
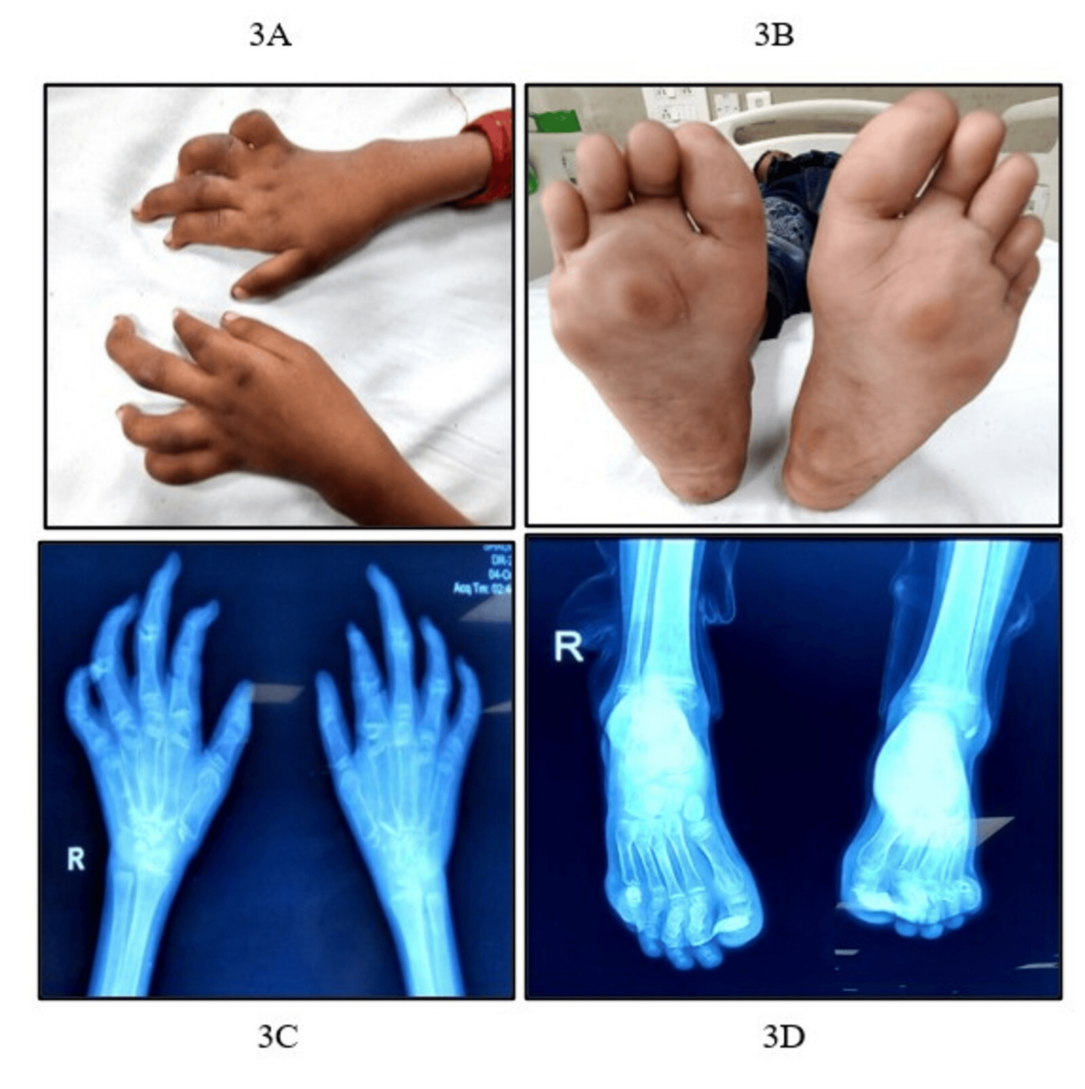
(A) Nodular swelling of MCP, PIP joints with contracture of PIP, DIP joints with wasting of thenar and hypothenar muscles. (B) Nodular swellings over bilateral plantar surface, hallux valgus, and contracture of the interphalangeal joints of the toes. (C) X-ray showing osteolysis of multiple carpal bones with flexion deformity of MCP, PIP, and DIP joints. (D) X-ray of the feet showing soft tissue swelling, diffuse osteopenia, and osteolysis of multiple tarsal bones with flexion deformity of the tarsometatarsal joints and IP joints. DIP, distal interphalangeal; IP, interphalangeal; MCP, metacarpophalangeal; PIP, proximal interphalangeal

Examination revealed swelling and flexion deformities of bilateral PIP and DIP joints of the hands (Figure 3A), knees, ankles, MTP joints, and interphalangeal (IP) joints of the feet, along with subcutaneous nodules over bilateral plantar surfaces, measuring 1.5x1.5x0.5 cm^3^ (Figure 3B). Spine and hip examinations were unremarkable. Investigations showed a hemoglobin level of 11.6 g/dL, WBC count of 3,211/mm³, and normal ESR, CRP, RF, anti-CCP, and ANA. Echocardiography was normal. Radiographs revealed osteolysis of multiple carpal bones with flexion deformity of MCP, PIP, and DIP (Figure 3C) and osteolysis of multiple tarsal bones with flexion deformity of tarsometatarsal and IP joints, along with diffuse osteopenia and prominent soft tissue swellings consistent with nodules (Figure 3D). Genetic testing confirmed a homozygous *MMP2* variant [c.556G>C; p.Gly186Arg], consistent with MONA syndrome. Details of the clinical features and investigations are described in Table 1.

**Table 1:** Clinical features and investigations of the patients CRP, C-reactive protein; DIP, distal interphalangeal joint; ECG, electrocardiogram; ESR, erythrocyte sedimentation rate; MTP, metatarsophalangeal joint; PIP, proximal interphalangeal joint

Characteristics	Patient 1	Patient 2
Gender	Male	Male
Consanguinity	No	No
Age at onset	6 years	4.5 years
Age at presentation	14 years	6 years
Family history	Sibling—similar history	No
Subcutaneous nodules	Yes	Yes
Skin lesions/hyperpigmentation	Yes	No
Hirsutism	No	No
Coarse face	No	No
Corneal opacity	No	No
Nose (bulbous/flat)	No	No
Gingival hypertrophy	No	No
Osteolysis of carpal/tarsal bones	Yes	Yes
Osteolysis of other bones	Phalangeal	Phalangeal
Contractures	PIP, DIP	PIP, DIP
Osteoporosis/osteopenia	Yes	Yes
Joint swelling	PIP, DIP, MTP, knee, wrist	PIP, DIP, MTP, knee, ankle
Joint stiffness	No	No
Reduced joint space	Yes	Yes
ESR (mm Hg)	12	15
CRP (mg/dL)	3.2	2.7
ECG	Normal	Normal
Congenital heart defect	No	No
Other important findings	Camptodactyly	Camptodactyly
Mutation	c.301C>Tp.Arg101Cys	c.556G>C; p.Gly186Arg

## Discussion

Methodology

Informed written consent was obtained from the parents of the patients described above. The search strategy for writing review articles proposed by Gasparyan et al. [15] was followed. ("Multicentric Osteolysis, Nodulosis, and Arthropathy" [MeSH Terms] OR "MONA syndrome" [All Fields] OR "Multicentric osteolysis" [All Fields] OR "Juvenile osteolysis" [All Fields] OR "Hereditary osteolysis" [All Fields] OR "Nodulosis" [All Fields]) OR ("Osteolysis" [MeSH Terms] OR "Bone Resorption" [MeSH Terms] OR "Arthropathy" [MeSH Terms] OR "Bone Diseases, Genetic" [MeSH Terms]) AND ("Matrix Metalloproteinase 2" [MeSH Terms] OR "MMP2 mutation" [All Fields] OR "MMP-2" [All Fields]) AND ("Review" [Publication Type] OR "Systematic Review" [Publication Type]) AND ("humans" [MeSH Terms]) AND ("English" [lang]) terms were used.

**Figure 4:**
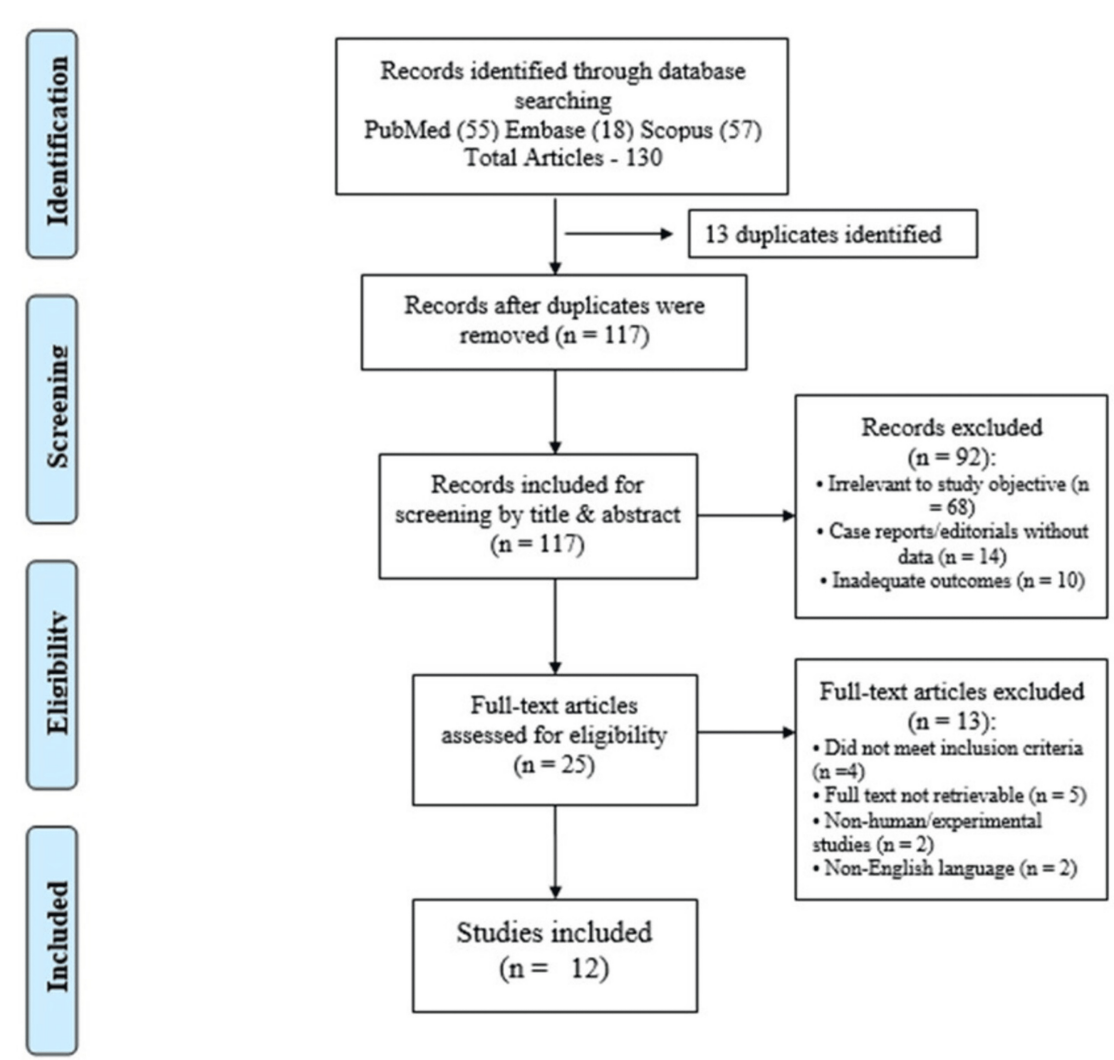
Flow chart of the search strategy

Priority was given to studies focused on MONA syndrome’s clinical features, imaging, genetics, treatment, and outcomes. Excluding inaccessible or irrelevant articles, key data - demographics, symptoms, and therapies - were extracted. A total of 130 articles were screened, out of which 12 articles were finally taken into consideration (Figure 4). This structured review enabled a comprehensive understanding of MONA’s clinical and genetic landscape.

Review of literature

There are few case reports and case series of MONA syndrome showing various clinical manifestations and novel mutations. Bhavani et al. discovered eight *MMP2* mutations in 13 people from 11 families - five unique and three known [2]. Detecting pathogenic variants enables carrier screening for relatives and allows prenatal or preimplantation genetic testing, offering early insights for families facing increased MONA risk. Due to the rarity of cases and presence of unique mutations, drawing a consistent genotype-phenotype correlation remains currently unreliable and challenging. Zankl et al. described an eight-month-old male child with coarse facial features and other known symptoms such as osteolysis and subcutaneous nodules [16]. Martignetti et al. reported four Saudi Arabian families with characteristic subcutaneous nodules, phalange osteolysis, and metacarpophalangeal and PIP contracture associated with osteopenia [17]. Other studies reported similar outcomes (Table 2).

**Table 2:** Review of Studies with different clinical, imaging, and mutations of MONA syndrome CRP, C-reactive protein; DIP, distal interphalangeal joint; ECG, electrocardiogram; ESR, erythrocyte sedimentation rate; MCP, metacarpophalangeal joint; MTP, metatarsophalangeal joint; PIP, proximal interphalangeal joint

Characteristics	Studies					
Studies	Martignetti et al. [17]	Zankl et al. [16]	Bhavani et al. [2]	Elsebaie et al. [18]	Mamadapur et al. [1]	Our case
Year	2001	2007	2016	2021	2024	2025
Study design	Case series	Case report	Case series	Case report	Case series	Case report
Country	USA	Switzerland	India	Egypt	India	India
Number of patients	35, 24 affected, 11 unaffected	1	13	2	3	2
Gender	Male and female, numbers not mentioned	Female	10 males, 3 females	male	1 Male, 2 females	Male
Consanguinity	Yes	No	Yes (67%)	Yes	1 patient	No
Age at onset (mean)	6 years	8 months	3 years	6.5 and 4 years	14 years	6 years
Age at presentation (mean)	14 years	8 months	8 years	2 years (both)	4.3 years	14 years
Family history	4 Saudi Arabian families	No	Yes (11 families)	No	Yes	Yes
Subcutaneous nodules	Yes	Yes	Yes	Yes	3 patients	Yes
Skin lesions/hyperpigmentation	No	No	No	No	1 patient	Yes
Hirsutism	No	No	Yes	No	2 patients	No
Coarse face	No	Yes	Yes	No	1 patient	No
Corneal opacity	No	No	No	No	No	No
Nose (bulbous/flat)	No	Yes	Yes	yes	No	No
Gingival hypertrophy	No	No	No	No	No	No
Osteolysis of carpal/tarsal bones	Yes	Yes	Yes	2 patients	3 patients	Yes
Osteolysis of other bones	Phalanges	Phalanges	Phalanges	Phalanges	Phalanges	Phalanges
Contractures	MCP, PIP	PIP, DIP	PIP	2 patients (PIP)	3 patients (PIP)	PIP, DIP
Large joints	Elbows, knees, shoulders	Knees, elbows, sacroiliac joints	No	No	No	Knee
Osteoporosis/osteopenia	Yes	Yes	Yes	2 patients	3 patients	Yes
Joint swelling	Yes	Yes	Yes	2 patients	3 patients	PIP, DIP, MTP, knee, wrist
Joint stiffness	No	Yes	No	1 patient	No	No
Reduced joint space	Yes	Yes	Yes	2 patients	3 patients	No
ESR (mm Hg)	NA	NA	Normal	NA	NA	12
CRP (mg/dL)	NA	NA	Normal	NA	0.3	3.2
ECG	Normal	NA	Normal	Normal	Normal	Normal
Congenital heart defect	No	No	Mild	Atrial septal defect: 1 patient	No	No
Other important findings	Frontal bossing, hypertelorism, Pathologic fractures of right humerus and femur (1 patient)	Abnormal gait was noted from the age of 13 months, transverse fracture of the proximal phalanx, High arched palate and epicanthal folds	NA	Patient 1: prominent forehead, hypertelorism, depressed nasal bridge, long philtrum, large ears, and a thick, protruding lower lip, without coarse facial features. Patient 2: triangular face with frontal bossing, thick eyebrows with partial synophrys, depressed nasal bridge, bilateral asymmetric ptosis, down-slanting palpebral fissures, strabismus, hypertelorism, thick protruding lower lip, low-set posteriorly rotated “bat” ears, and a relatively thin chin.	2: subcutaneous swelling over both feet. 1: camptodactyly, subluxation of 1-5 MTP joints, ankylosis of carpal bones	Camptodactyly
Mutation	Two family-specific mutations R101H, Y244X	Two mutations in exon 2 (302 G>A) (R101H); the mother is also having R101H mutation and a 1-bp deletion (1357delC) in exon 8	Five novel mutations: two deletions (p.S304Pfs*115 and p.N430Tfs*68), two nonsense variants (p.C102* and p.Y263*), and one missense variation (p.G410V)	c.302G>A p. (Arg101His), c.40del p.(Leu14fs*)	1^st^: c.301C>T p(Arg101Cys). 2^nd^: c.380G>A p.Arg127Lys. 3^rd^: c.380G>A p.Arg127Lys.	c.301C>T p.Arg101Cys. c.556G>Cp.Gly186Arg.

A five-year-old boy diagnosed with MONA syndrome and associated congenital heart defects, reported by Castberg et al., showed ongoing bone loss despite treatment. This example demonstrates a possible cardiac relationship in MONA, recommending routine heart examinations for all affected individuals [19].

Discussion

Torg syndrome, Winchester syndrome, Frank-Ter Haar syndrome, and MONA are currently regarded as a single spectrum of multisystem illnesses manifesting overlapping features involving the skin, joints, and bones. Homozygous mutations in the *SH3PXD2B* and *MMP14* genes cause Frank-Ter Haar syndrome and Winchester syndrome, respectively, whereas *MMP2* gene mutation causes Torg and MONA syndromes [3,16]. Compared to Winchester syndrome, Torg syndrome exhibits milder phenotypic characteristics, such as the lack of marked coarse facial features, eye opacities, cardiac manifestations, and pronounced vertebral involvement [7].

Both of our patients had typical subcutaneous nodules, osteolysis of phalanges, camptodactyly, and hyperpigmented skin lesions. The symptoms may be confused with those of hyaline fibrosis syndrome, mucopolysaccharidosis, multicentric carpal osteolysis with or without nephropathy, and juvenile idiopathic arthritis, which could cause a delayed diagnosis. Here are two examples, presenting in childhood with swollen joints, nodules, and progressing arthropathy. Most children with MONA appear healthy at birth. The illness typically manifests in the first few years of life as arthritic symptoms, such as hands and feet swelling, contractures, or joint pain. Arthropathy is persistently progressive [2]. Males are affected more commonly, as reported in the literature (Table 2). Consanguinity is found in around 67% of the patients [2]. Fever is rare. Later in the condition, individuals have coarse facial characteristics and gum hypertrophy. Most of them grow solid, palpable nodules under the skin. Numbers and sizes of the nodules gradually grow with time. Osteolysis of underlying bones can cause joint laxity in the hands and feet [2].

Typically, there are no biochemical signs of inflammation [2]. This illness is characterized radiographically by lysis of the carpal and tarsal bones. Other hand and foot bones, such as the metacarpals, metatarsals, and phalanges, are frequently affected. These bones have inadequate modelling, resulting in a wide, uneven look. Bone cortices appear thin. In older children, osteoporosis or osteopenia affects the entire skeletal system.

Genetic testing for MONA syndrome was performed using next-generation sequencing targeting the *MMP2 *gene, followed by Sanger sequencing for variant confirmation. Identified variants were interpreted according to ACMG guidelines, integrating population frequency, in silico predictions, and literature evidence to establish pathogenicity and ensure diagnostic accuracy for this rare osteolytic disorder.

Treatment for MONA remains largely supportive. Calcium, vitamin D, and bisphosphonates may alleviate bone pain and enhance mineralization [20]; however, joint mobility is frequently unaffected. Steroids and immunosuppressants have no benefit. Early intervention, physical therapy, and adaptive assistance can help manage symptoms; however, surgery for contractures provides uneven relief [18].

These two cases highlight the typical clinical and radiological manifestations of MONA syndrome, which will help the primary physician suspect and make an early referral to rheumatologists or medical genetics. Genetic testing is vital in diagnosing MONA syndrome by identifying the *MMP*2 mutation, differentiating it from similar inflammatory conditions, and avoiding inappropriate therapies. It further aids in carrier screening and genetic counselling and enhances the understanding of genotype-phenotype relationships to improve patient care and management. Definitely more case reports and case series are needed to have a better understanding of novel mutations and treatment options.

## Conclusions

MONA syndrome is a rare, progressive skeletal disorder with significant diagnostic and therapeutic challenges. Clinical findings and genetic analysis confirm the diagnosis, and treatment is supportive. Increased awareness among clinicians, continued case reporting, and research into targeted gene therapies are essential to enhance understanding and management of this debilitating condition.
